# Safety and Acceptability of a Natural Language Artificial Intelligence Assistant to Deliver Clinical Follow-up to Cataract Surgery Patients: Proposal

**DOI:** 10.2196/27227

**Published:** 2021-07-28

**Authors:** Nick de Pennington, Guy Mole, Ernest Lim, Madison Milne-Ives, Eduardo Normando, Kanmin Xue, Edward Meinert

**Affiliations:** 1 Ufonia Ltd Oxford United Kingdom; 2 Centre for Health Technology University of Plymouth Plymouth United Kingdom; 3 Imperial College London London United Kingdom; 4 University of Oxford Oxford United Kingdom; 5 Department of Primary Care and Public Health Imperial College London London United Kingdom

**Keywords:** artificial intelligence, natural language processing, telemedicine, cataract, aftercare, speech recognition software, medical informatics, health services, health communication, delivery of health care, patient acceptance of health care, mental health, cell phone, internet, conversational agent, chatbot, expert systems, dialogue system, relational agent

## Abstract

**Background:**

Due to an aging population, the demand for many services is exceeding the capacity of the clinical workforce. As a result, staff are facing a crisis of burnout from being pressured to deliver high-volume workloads, driving increasing costs for providers. Artificial intelligence (AI), in the form of conversational agents, presents a possible opportunity to enable efficiency in the delivery of care.

**Objective:**

This study aims to evaluate the effectiveness, usability, and acceptability of Dora agent: Ufonia’s autonomous voice conversational agent, an AI-enabled autonomous telemedicine call for the detection of postoperative cataract surgery patients who require further assessment. The objectives of this study are to establish Dora’s efficacy in comparison with an expert clinician, determine baseline sensitivity and specificity for the detection of true complications, evaluate patient acceptability, collect evidence for cost-effectiveness, and capture data to support further development and evaluation.

**Methods:**

Using an implementation science construct, the interdisciplinary study will be a mixed methods phase 1 pilot establishing interobserver reliability of the system, usability, and acceptability. This will be done using the following scales and frameworks: the system usability scale; assessment of Health Information Technology Interventions in Evidence-Based Medicine Evaluation Framework; the telehealth usability questionnaire; and the Non-Adoption, Abandonment, and Challenges to the Scale-up, Spread and Suitability framework.

**Results:**

The evaluation is expected to show that conversational technology can be used to conduct an accurate assessment and that it is acceptable to different populations with different backgrounds. In addition, the results will demonstrate how successfully the system can be delivered in organizations with different clinical pathways and how it can be integrated with their existing platforms.

**Conclusions:**

The project’s key contributions will be evidence of the effectiveness of AI voice conversational agents and their associated usability and acceptability.

**International Registered Report Identifier (IRRID):**

PRR1-10.2196/27227

## Introduction

### Background and Rationale

#### Clinical Problem

In the United Kingdom, aging population is causing an increased demand for health care services that is exceeding clinical capacity [1]. With staff pressured to deliver high-volume workloads, the resulting burnout crisis is increasing costs for health care providers. Demand has been further exacerbated by the COVID-19 pandemic, as the widespread cancelation of elective care has created a large backlog of clinical work [2]. However, a large proportion of this clinical work is taken up by highly repetitive and low-skill tasks, preventing staff from working *at the top of their license*. Therefore, there is a need to improve the efficiency in the delivery of care and to collect data that can be analyzed to support routine improvement and optimization through the automation of routine clinical interactions.

One area of care where improved efficiency is urgently needed is cataract surgery. Cataract surgery is the most common operation in the National Health Service (NHS), with approximately 450,000 procedures conducted per year [3]. COVID-19 has caused record delays in receiving planned surgeries [4], and the average wait time for cataract surgery was approximately 2.5 months [5]. The aging population will also have a significant impact on the number of patients with cataracts, which is expected to double between now and 2050 [6]. The cataract pathway is also an ideal case for optimization because there is little variability and high levels of patient safety; the most significant complication (endophthalmitis) occurs in fewer than 1 in 1000 cases [7]. To address this clinical need, this study aims to collect data on the effectiveness, usability, and acceptability of an artificial intelligence (AI) natural language assistant for delivering cataract surgery follow-up checks.

#### Current System and Its Limitations

Similar to most operations, cataract surgery requires a postoperative check to monitor for complications and assess success. This has historically been performed with a face-to-face visit; prior to the COVID-19 pandemic, this was the standard procedure for 72% of NHS Trusts [8]. However, this postoperative system has a high operational demand and is not always necessary; a recent ophthalmology Getting It Right First Time (GIRFT) report stated that a hospital review of cataract surgery patients is not required, as long as follow-up arrangements are in place [8]. In the current context of the COVID-19 pandemic, face-to-face visits also pose a high risk for virus transmission due to the proximity of patients and clinicians.

Although this project focuses on cataract surgery follow-up, the underlying platform will be applicable to a wide range of routine clinical tasks. The need for an effective automated tool is especially great, as a *new normal* of widespread remote clinical care has been established in the wake of COVID-19.

#### User Needs

Using AI-enabled automation is a novel approach for patient care, and patient perspectives are mixed [9,10]. Research on cataract surgery patients during the development of this solution found that although many patients stated that they would prefer a clinician to provide follow-up, this opinion was contingent on the quality of the system and service [11]. Most patients rated the solution highly on simplicity and ease of use and expressed appreciation for the additional convenience of a telephone follow-up [11].

The patients also suggested that the AI system might be less rushed than a clinician and would have the benefit of freeing up nurses’ time for other clinical work. This is supported by internal company research on the clinical outcomes of 300 cataract surgery follow-up calls, which identified that only 10% (30/300) of patients telephoned by expert clinicians were determined to need a face-to-face review. Therefore, an automated telephone follow-up could significantly reduce the number of clinical appointments necessary to deliver postoperative care.

### Solution Overview

#### General Description

The solution developed to improve clinical efficiency for cataract surgery follow-up is a natural language, voice telemedicine conversation delivered to patients via telephone calls. For the patient, this is intended to be no different than a regular telemedicine consultation with a doctor or nurse; it does not require the download of an app, the provision of a device, or any training. This is important because the populations that consume the majority of health care services (older adults and socioeconomically disadvantaged populations) tend to be relatively more digitally disenfranchised.

The solution, a natural language AI assistant called Dora agent: Ufonia’s autonomous voice conversational agent, has been developed by Ufonia Limited. By the start of the clinical testing described in this proposal, validation with Ufonia’s development partners at Buckinghamshire Healthcare NHS Trust will be complete, and Dora will be at Technical Readiness Level (TRL)5. By the end of this study, the solution will be at TRL6, which has been demonstrated in a relevant clinical setting.

#### Key Features

Dora uses a variety of AI technologies to deliver the patient follow-up call, including speech transcription, natural language understanding, a machine learning conversation model to enable contextual conversations, and speech generation. Together, these technologies cover the input, processing and analysis, and output needed to maintain a natural conversation. Dora is currently delivered via a telephone connection as a real-time, stand-alone system.

### Benefits of Proposed Solution

#### For Digitally Enabled Care

The solution is aligned with the aims of NHS to improve efficiency and increase digitally enabled care [8,12]. Cataract surgery follow-up provides a good launching point for further development of digital solutions, as it typifies many routine telemedicine care processes that could be similarly automated.

#### For Health Care Staff

The solution is expected to have significant benefits for health care staff by reducing their clinical burden. Preliminary company research suggests that Dora will reduce the number of patients who require follow-up with a clinician by approximately 80%. This reduces the type of high-volume, repetitive task that contributes to burnout and allows clinicians to be redeployed to higher-value activities, where their skills, insight, and empathy can be best used. In addition, telephone follow-up reduces the risk of COVID-19 transmission and frees up hospital space to help meet the increasing patient demand.

#### For Patients

The system also has several benefits for patients. Dora will provide a reliable and consistent safety net after surgery. Patients will be able to ask questions about their recovery (such as when to drive, swim, and stop taking eye drops) just as they would with a human clinician. The system is convenient because it does not require them to travel to the hospital and can take place at the time or duration that suits them. Reducing the number of in-person follow-ups will allow clinicians to perform other clinical activities, making patients more likely to receive timely care for their initial cataract surgery or for other conditions. This will be evaluated as part of the study by examining the number of surgeries conducted.

### Aims and Objectives

The purpose of this study is to evaluate the evidence for clinical safety, study design feasibility, usability, acceptability, satisfaction, appropriateness, and cost-effectiveness of the autonomous cataract follow-up call system (Dora) for detecting patients that require further assessment. The primary aims are to establish preliminary evidence that Dora is safe, to evaluate its sensitivity and specificity, and to determine what can be learned to improve its design for future studies.

To achieve these aims, there are 5 key objectives:

1. To establish baseline rates of efficacy for Dora’s detection of patients requiring further assessment
2. To evaluate patient acceptability of an autonomous call in comparison with existing standards of care
3. To evaluate the cost-effectiveness of autonomous calls in comparison with existing standards of care
4. To capture conversational data to train future versions of the system
5. To capture data and assess study feasibility to inform the development of future trials

### Research Questions

On the basis of these aims and objectives, 4 main research questions were defined to guide the project:

1. What are the factors impacting the effectiveness of Dora’s conversational call follow-up to determine patients who require further assessment?
2. Can Dora sufficiently support conversation and patient engagement to collect the data needed to allow Dora’s computational capabilities to perform an accurate assessment?
3. What are the perceived benefits and barriers of using conversational agents for patient follow-up?
4. Is Dora more cost-effective than existing standards of care?

## Methods

### Study Design

Using an implementation science construct, the study will be a phase 1 pilot study to develop evidence regarding the feasibility, acceptability, and potential effectiveness of Dora and to identify factors influencing effectiveness. The study will last for 18 months: 6 months of evaluation and intervention refinement, 9 months of implementation and follow-up, and 3 months of postevaluation analysis and write-up.

### Research Participants

The study population will incorporate two clinical sites: the Imperial College Healthcare NHS Trust and the Oxford University Hospitals NHS Trust. The population at the Imperial College site is drawn from the North West London Collaboration of Clinical Commissioning Groups (CCGs) and is densely populated, highly diverse, highly mobile, and relatively young [13]. Black, Asian, and other minority ethnic groups make up 37% of the resident population in the West London CCG [14], and 20% of London residents do not speak English as their first language [15].

In contrast to London, the Oxfordshire population is less diverse but more rural [16]. Although it is growing in ethnic diversity, residents are primarily of a White British background; in 2011, approximately 84% of Oxfordshire residents identified as White British (compared with the national average of approximately 80%) [17]. It was also found that 16% of the Oxfordshire population did not speak English as their main language (compared with the national average of 8%) [18]. In terms of age, the population of Oxfordshire is similar to the national profile, particularly for older ages [19].

### Recruitment

Recruitment will take place at sites in the Imperial College Healthcare and Oxford University Hospitals NHS Trusts that conduct cataract surgeries. Study information will be shared with cataract surgery patients at their initial visit (and via post or telephone call for patients who are delayed by COVID-19 or cannot visit in person). Informed consent will be obtained at the time of preassessment (in-person or virtually depending on the current COVID-19 guidelines). In the discharge lounge following surgery, patients who have consented to participate will be given further information to remind them about the call from Dora. This work will be performed by a dedicated research nurse at each site.

### Study Duration and Follow-up

This study will conduct telemedicine calls with patients in addition to their standard of care. Patients will receive their call from Dora between 25 and 27 days post surgery. The evaluation of Dora in two different NHS Trusts will help demonstrate that Dora can be integrated into different pathways. It will also provide a range of sensitivities to input into the health economic models to determine whether Dora provides cost-effectiveness in different settings.

### Procedure

#### Overview

Although patients typically have cataracts in both eyes, procedures are typically performed on one eye at a time in the United Kingdom. As this is a pragmatic study, patients undergoing either first or second eye surgery will be recruited. This should be in a balanced proportion due to the random nature of operating timing, but differences between groups will be examined in the posthoc analysis. Given that the usual interval between cataract surgeries is more than 6 months, it is unlikely that the same patient will be recruited twice in the study. If there are any cases where the same patient is recruited, they will be excluded from participating for the second time.

#### Dora Call

The call that patients will receive from Dora will include several conversational elements:

- Greeting and introduction
- Identification of patient
- Cataract follow-up questions
- Patient’s queries
- Decision
- Questions about acceptability
- Closure of call

The entire conversation will be supervised by an expert clinician (an ophthalmology research fellow). This clinician will be able to interrupt the call at any point if the system fails, the patient struggles to interact with it, or Dora does not collect sufficient information from the patient.

The cataract follow-up questions will classify 5 key symptoms: redness, pain, reduced vision, flashing lights, and floaters. Both Dora and the supervising clinician (masked to each other) will independently indicate for each symptom, whether the symptom is:

1. Absent (eg, no pain)
2. Present but not clinically significant (eg, mild gritty sensation)
3. Present and clinically significant (eg, deep and persistent pain)
4. Insufficient information for classification

Issues identified in response to any of these questions will prompt the need for a face-to-face review. The complexity of the model comes from evaluating the exact nature of these symptoms, for example, distinguishing between improving redness in the corner of the eye (due to the local anesthetic injection) and widespread redness that has progressed, which may represent infection. The conversational nature of the model enables it to ask patients further questions to clarify their responses and assess the significance of the reported symptoms.

If clarifying points are necessary for the clinician to make a decision, Dora will enable the clinician who will ask the necessary questions, and then record updated assessments for symptoms and overall management before handing back to Dora for frequently asked questions and patient evaluation.

If the call is uninterrupted, Dora will make a decision about the patient’s management plan. Once the cataract follow-up questions have been completed, the clinician will also set their own decision, masked to the decision made by the system (Table 1). For this project, the supervising clinician’s decision, which is made based on the information from the call with Dora’s decision masked, is considered the gold standard for evaluation. For each patient, the final decision regarding the management plan can be as follows:

1. Discharge (and/or add to waiting list for second eye cataract surgery and/or continue follow-up as previously planned in the ophthalmology clinic)
2. Eye casualty review within 1 week
3. Same day eye casualty review

**Table 1:** Outcome communicated to the patient at the end of the call depending on the blinded decision of Digital Online Recording Automation agent: Ufonia’s autonomous voice conversational agent and the clinician.

Clinician decision	Outcome
Does not need review	No review
Needs review	Review
Insufficient information	Interrupt call

If the patient needs review, they will either be seen at the scheduled appointment the following day or by the clinical fellow the following day, if an appointment is not scheduled (at Oxford or if pathways change due to COVID-19). If they are found to have a complication, they will enter the existing NHS care pathways as they would with typical care. If the complication is deemed urgent by the clinician, the patient will be seen the same day.

### Theoretical Framework

The evaluation of baseline efficacy, sensitivity and specificity, feasibility of study design, acceptability, and usability shall be conducted using the following scales and theoretical models and frameworks:

1. The system usability scale [20]
2. Assessment of Health Information Technology Interventions in Evidence-Based Medicine Evaluation Framework [21]
3. The telehealth usability questionnaire [22]
4. Long-term adoption and suitability for further trials will be evaluated using the Non-Adoption, Abandonment and Challenges to the Scale-up, Spread, and Suitability framework [23]

#### Data Collection

The key element of the conversation that this study will assess is Dora’s ability to make *correct* decisions about whether review is necessary. For this project, the correct decision is that which the supervising clinician makes based on the information they hear. Therefore, the key data to be collected are Dora’s and the clinician’s decisions.

The call will also collect data about the individual patient’s condition. The follow-up questions are aligned with existing cataract patient-reported outcome measures so that they meet the needs of responsible clinicians and can be used to populate the National Ophthalmology Database [3].

The usability and acceptability of the system will be assessed through automated questions at the end of each call. In addition, a sample of patients with good and poor experience will be approached to have a more in-depth interview (as detailed in the *Ethics* section).

#### Data Analysis

The primary analysis will be the calculation of a kappa statistic of interobserver (Dora and clinician) reliability of the decision made. In addition, the outcome of the assessment will be compared with the *real* complication rate determined by any face-to-face assessments. This will be established by the identification of any patient presentation within 60 days after the last call. Given the specialist nature of ophthalmology services, it can be assured that patients will present through the eye casualty services offered by each site. This analysis will provide baseline sensitivity and specificity data for use in preparing subsequent evaluations of efficacy.

The usability and acceptability questions delivered at the end of the call will be analyzed quantitatively based on the scales’ scoring criteria; for instance, a score above 80 on the system usability scale is generally considered to indicate an above average user experience [24]. The interview recordings will be transcribed and assessed using thematic analysis.

#### Bias

To prevent participation bias, there will be no exclusion of participants who are willing to participate in the study, unless they have a relationship with any of the researchers associated with the study (to avoid conflicts of interest). To address unconscious bias or other forms of interview recruitment issues, interview participants will be selected randomly by a computer script of consented participants. A quantitative analysis of the use experience will include all participants to avoid recruitment bias. Participants’ levels of education will be recorded to note possible ways in which education impacts intervention use.

It is vital to maintain the integrity of the study and avoid any commercial influence on the results. Therefore, a research contract will be established between Ufonia and the academic research partners, enabling unrestricted right of publication of nonconfidential information and independence of study implementation.

#### Risks

The COVID-19 pandemic has raised potential issues for recruitment for the study. To address this risk, an ethics submission will enable remote informed consent if restrictions prohibit face-to-face contact.

Risks regarding the system include it failing to detect patients who require urgent clinical review, which will be mitigated by having an expert clinician supervise the call in real time and intervene if needed. The risk of lack of trust in the system has been mitigated by an extensive cocreation process for Dora, which focuses on user-centered design.

The interviews are scheduled to take place for 40 to 60 min, to mitigate time risk to participants. The nature of interview questions avoids areas of cultural or psychological sensitivity and is purely focused on the impact of the intervention. To control any potential perceived issues in this area, participant confidentiality is protected using data protection procedures that are compliant with the General Data Protection Regulation (GDPR) [25].

### Ethical Considerations

#### Study Governance

Research Ethics Committee permission has been granted. This manuscript provides an overview of the study approach, but it will be subject to further revision before ethical submission. An independent study steering board composed of the academic principal investigator (PI), co-academic PI, a study researcher, a member of the public, and an external researcher will meet every 2 months to review progress against the study plan and to assure study ethics are being followed. Reports shall be distributed for review by the project team for action. To ensure the validity, reliability, and transferability of the study findings, the Consolidated Standards of Reporting Trials, Standard Protocol Items: Recommendations for Interventional Trials, and Consolidated Criteria for Reporting Qualitative Studies guidelines, including additional AI-related extensions, will be followed and recorded in the study protocol.

#### Informed Consent

British educational research association guidelines have been followed for voluntary informed consent, use of methods, and university policies in the event that there are issues in delivery [26]. Before completing informed consent, participants will be given information that fully describes the process of the study, including why their participation is necessary, how their data will be used, and who the results will be reported to. As many patients are understandably concerned about how their data will be used, data management will be explained in detail as part of the consent process. It will also make clear their right to withdraw from the study at any time and have their data destroyed. Patients will also be asked to separately consent for their data to be used to further train the conversational systems. Declining to share this conversation data will not affect patients’ participation in the study or their clinical care.

#### Data Management

The Ufonia system stores patient identifiable data as part of the clinical record. Explicit consent is obtained from patients to use these data in ongoing development. The solution is in compliance with the GDPR [25] and is being built to meet specific NHS regulations. For the proposed study, the organizations involved (including each Trusts’ Data Protection Officer and Caldicott Guardian) will undertake a Data Protection Impact Assessment and, where needed, create information sharing agreements to ensure compliance with relevant data protection regulations. Ufonia will act as the data processor, and individual hospitals will remain the data controllers.

During the study implementation, each participant will be given a unique identifier. The primary key between the unique ID and participant will be securely held and given to the participant as a reference ID. Data will be analyzed using the unique IDs; the primary key is only maintained to enable participants to withdraw their data from the study. If such a request is made before data aggregation or publication, all of their corresponding data and files will be destroyed.

Audio recordings, transcriptions, and metadata about the calls will be securely stored in UK datacenters with strict role-based access control. The transcription service will only have reference to the unique IDs, and the audio recording will be reviewed by the PI to remove any identifying information before being shared. Patient identifiable data will not be sold to any other party and will not be shared with any organization unless they are a partner in the study and have an appropriate information sharing agreement in place. In accordance with GDPR requirements, records of consent will be kept for 3 years after the publication of the final study results [25].

## Results

The evaluation is expected to show that conversational technology can conduct an accurate assessment and that it is acceptable to different populations with different backgrounds. In addition, the results will demonstrate how successfully the system can be delivered in organizations with different clinical pathways and can integrate with their existing platforms.

## Discussion

### Overview

This project will establish a strong foundational evidence for the use and wider deployment of a novel application of AI technologies. The platform has the potential to transform the delivery of care across multiple clinical pathways by reducing costs, increasing capacity, and improving the convenience and experience for patients and professionals.

The key outputs from this project will provide the following:

1. Safety and preliminary efficacy data for regulatory approval.
2. Proposed structure and implementation model for further clinical trials.
3. Health economic data to support wider roll-out and ongoing evaluation.
4. Results submitted for peer-reviewed publication.

### Limitations

A limitation of Dora is that, at present, patients with cognitive difficulties or hearing impairment or non-English speakers will not be able to use the system, facing the same limitations they currently do with human telemedicine services.

A limitation of the study is that a direct comparison of the number of issues identified during routine follow-up cannot be made between the Oxford and Imperial sites. This is because the Oxford University Hospitals NHS Trust does not proactively review patients, instead relying on them to present themselves to their eye casualty service. This introduces a potential risk for missed complications if Dora does not decide that a review is needed. However, this risk is minimized by expert clinician oversight and is a part of the current standard of care at the Oxford University Hospitals NHS Trust.

## Appendix

Multimedia Appendix 1 Peer-review feedback from NHS.

## Abbreviations

AI: artificial intelligence
CCG: Clinical Commissioning Group
GDPR: General Data Protection Regulation
NHS: National Health Service
OUH: Oxford University Hospitals
PI: principal investigator
TRL: Technical Readiness Level
