# Supplementary material for: Safety and Acceptability of a Natural Language Artificial Intelligence Assistant to Deliver Clinical Follow-up to Cataract Surgery Patients: Proposal
Source: JMIR Res Protoc. 2021 Jul 28;10(7):e27227. doi: 10.2196/27227 (PMC8367096; doi:10.2196/27227)
Supplement: Multimedia Appendix 1 [file resprot_v10i7e27227_app1.pdf]

ACCELERATED ACCESS COLLABORATIVE

NIHR | National Institute for Health Research

NHSX

Artificial Intelligence in Health and Care Award Peer Review

Reference Number	AI_AWARD01852
Project Title	Autonomous Telemedicine - Cataract Surgery Follow-up at Two NHS Trusts
Lead Applicant	Dr Nick de Pennington
Host Organisation	Ufonia Limited
Contract Costs	£503,524.00
Reviewer Number	1

Table Of Contents

1. Reviewer Expertise
2. Relevance of the proposed research
3. Quality of the proposed work
4. Strength of the research team
5. Impact of the proposed work
6. Value for money
7. Involvement of patients and the public
8. Additional Comments

1. Reviewer Expertise

**Please indicate the nature of your expertise by clicking on the appropriate tick box(es) below:**
Clinician in the same/a very similar field

If the tick boxes above do not adequately capture the nature of your expertise, please briefly provide details in the box below (or use it to give us more detail about your expertise if you wish):

2. Relevance of the proposed research

i) How relevant and important is the proposed research to the priorities and needs of the NHS, and does it offer a health/healthcare solution with demonstrable benefit to patients?<br>ii) Does the application demonstrate an awareness and understanding of previous relevant research or developments in this area?<br>iii) To what extent does the proposed research add distinct value or advance existing knowledge in this area, taking into account wider ongoing or completed research?
The application is very well written and details provided are excellent.<br><br>i) It's very relevant. Cataracts are important blinding causes and it's reversible - the sooner you remove it by surgery, the sooner the patient will be back in his life and work activities and less chances for complications.<br><br>ii) yes, very much.<br><br>iii) It adds value during the COVID19 time and evaluate the real need patients have to attend an appointment. But without the pandemic and life getting back to people being able to be outside without a problem, I as a surgeon and most of the patients interviewed by them, prefer a clinician to provide follow up cataract surgery appointments. If the patient is recovering well then this complementary tool would be very interesting.

3. Quality of the proposed work

**Research Design**<br>i) How appropriate is the research design in relation to the stated objectives?<br>ii) To what extent is the proposed design and methodology for all elements of the research well defined, appropriate, valid and feasible within the timeframe and resources requested?<br>iii) What are the strengths and weaknesses of the research design as proposed?<br>iv) To what extent does the research show originality and innovation?
i) It seems appropriate.<br><br>ii) They match well.<br><br>iii) **Strengths:** it is interesting to have a machine to answering questions that surgeons would, when are questions the surgeons already told on the appointment and they forgot or news doubts patients have when they are home and need the answer before they are able to speak with their surgeon - as they say "a safety-net after surgery".<br><br>**Weaknesses:** It gives the impression that surgeons want to relive the burden of talking to their patients ("DORA will reduce the clinical burden on staff by reducing the number of patients who require follow up with a clinician by approximately 90%") - It believe getting in touch with their patient before and after surgery is a higher value activity and should not be taken for granted or given to someone else. It is a great part of the patient's recovery and it is what gives the patient confidence the procedure will have a good outcome (outcome as the patient will recover well and outcome as the surgeon's positive feedback - best business card a physician/surgeon can have are their patients stating they are good). Another thing is: if the entire conversation will be supervised by an expert clinician, how will they free up this clinician to a higher value activity? (It was not clear to me).<br><br>iv) It is the only one I have heard of.

Has the research been designed with reference to an appropriate review of the existing literature?
Yes

4. Strength of the research team

i) How well are the roles of the team members described? Is the overall team well coordinated?<br>ii) On the basis of track record in relevant areas, how qualified are the applicants to undertake the work using the methodologies proposed?<br>iii) To what extent does the research team have the necessary breadth and depth of expertise to deliver the proposed work (*e.g.* as judged by publication output and previous research funding)?<br>iv) How could the strength of the research team be improved?<br>v) If the lead applicant is inexperienced, does he/she have appropriate support (*e.g.* from their organisation and/or more senior colleagues) to deliver the work plan?
i) Roles are well and overall well coordinated.<br><br>ii) I have read and re-read about their qualification but I cannot find a Cataract surgeon.<br><br>iii) They are enough breadth and depth of expertise in their field and in AI field but I am not sure if they are expertise in cataract surgery.<br><br>iv) I would have a cataract surgeon with senior experience on the team.

v) Lead applicant and his team seems experienced digital innovation in Medicine but I cannot find anyone specialist in cataract surgery.

5. Impact of the proposed work

Dissemination, outputs and anticipated impact
NIHR aims to fund research that has the potential to be of significant benefit to the NHS, patients and the public. To support this, the applicants should consider how they will achieve impact from the outset. This helps them to identify potential impact and beneficiaries, and plan processes by which the research can directly, or incrementally over time, lead to change.

i) Have the applicants clearly expressed the problem and outlined how this research contributes towards a solution? Are the planned outputs appropriate and sufficient?
ii) Is it clear from the application who or which groups (including, as appropriate, healthcare planners, clinicians, patients and/or policy makers) are expected to benefit the most, how they will be engaged and communicated with, and if appropriate methods of engagement and dissemination are planned?
iii) Have the applicants set out appropriate activities and resources to achieve their impact goals? Is there a realistic trajectory and estimate of timescales for the benefit to reach patients/ public/ health and care services? Are there clear connections between outputs, engagement processes and impact goals?
iv) Have the applicants clearly considered what follow-on support they might need to generate or upscale impact, and how they might leverage further investment?
v) Have the applicants sufficiently and correctly identified any potential barriers they might face (e.g. IP, regulatory and acceptability) and have they properly considered how these may be overcome?
vi) Have the applicants clearly and realistically outlined the anticipated impacts, the likely scale of these impacts (both in the shorter and longer terms), and the sequence in which the impacts might occur?

i) and ii) Yes to all questions.

iii) I am not sure.

iv) and v) It seems so.

vi) I am not sure - I tend to think that patients can even join the project but that the project will not decrease the frequency of appointments. Surgical patients need attention and present care that I am not sure this project will be able to provide.

6. Value for money

i) Taking into consideration the costs associated with undertaking the research, is there sufficient justification for the resources requested? Are all costs essential for the work proposed?
ii) Are appropriate resources set aside for patient and public involvement - including plans for a training and support budget?
iii) Where relevant, are resources to support impact - other than those for public and patient involvement - included and appropriate?
iv) Taking into account the total cost of the research, including the NHS costs, to what extent does the research provide value for money?
v) If required, are funds requested for NHS support and treatment costs appropriate and justified?

i) I honestly don't think so for both questions.

ii) Yes.<br><br>iii) I don't know.<br><br>iv) I believe not.<br><br>v) I haven't found them if there are.

7. Involvement of patients and the public

**Was there any patient and public involvement in the application?**	Yes

i) What is your assessment of the patient and public involvement (if any) in the development of the application, including involvement in: Identifying the research topic; prioritising the research questions; preparing the application (e.g. contributing to the research design); and identifying potential impact?<br>ii) What is your assessment of any proposed plans for patient and public involvement throughout the life of the research? Can you identify particular strengths, weaknesses and/or areas for improvement?
i) In my assessment, the patient and public involvement provides a good feedback in how the project is going to be accepted and what adjustments in the interface it can be necessary. They can contribute to the research design that will be able to fit them better and identifying potential impacts and providing feedback - as it already have in this project.<br><br>ii) I am not sure.

8. Additional Comments

If you have any additional comments, not covered by the sections above, please provide them below.
No additional comments.

ACCELERATED ACCESS COLLABORATIVE

NIHR | National Institute for Health Research

NHSX

Artificial Intelligence in Health and Care Award Peer Review

Reference Number	AI_AWARD01852
Project Title	Autonomous Telemedicine - Cataract Surgery Follow-up at Two NHS Trusts
Lead Applicant	Dr Nick de Pennington
Host Organisation	Ufonia Limited
Contract Costs	£503,524.00
Reviewer Number	2

Table Of Contents

1. Reviewer Expertise
2. Relevance of the proposed research
3. Quality of the proposed work
4. Strength of the research team
5. Impact of the proposed work
6. Value for money
7. Involvement of patients and the public
8. Additional Comments

1. Reviewer Expertise

**Please indicate the nature of your expertise by clicking on the appropriate tick box(es) below:**
Researcher in a broadly related field

If the tick boxes above do not adequately capture the nature of your expertise, please briefly provide details in the box below (or use it to give us more detail about your expertise if you wish):
Expertise in digital health technologies, user experience, patient safety.

2. Relevance of the proposed research

i) How relevant and important is the proposed research to the priorities and needs of the NHS, and does it offer a health/healthcare solution with demonstrable benefit to patients?<br>ii) Does the application demonstrate an awareness and understanding of previous relevant research or developments in this area?<br>iii) To what extent does the proposed research add distinct value or advance existing knowledge in this area, taking into account wider ongoing or completed research?
i) This research is important and timely. Particularly at a time where social distancing is limiting what is possible to do efficiently face-to-face, this kind of solution is essential to deliver a high standard of patient care, but similar solutions are needed long-term. There is little discussion in the bid of how the findings might generalise to other conditions or to other Trusts, but I would expect the learning from this project to be quite widely applicable.<br><br>ii) The discussion of previous relevant research is fairly limited in this bid, but that reflects its origins and directions. The bid is for translational research that should have an immediate impact on practice, and there is more extensive discussion of alternative products than of underpinning research. In this particular case, that seems the best approach given the TRL of the proposed studies. There are areas that could have been discussed in greater depth (e.g., conversational agents, decision support systems, patient safety, user experience), but in-depth discussion of all these areas would have resulted in a book rather than a research proposal.<br><br>iii) The proposed research adds distinct value in its integration of multiple areas of existing knowledge to address

an immediate practical problem (or opportunity) in healthcare delivery. Publications are planned, which should distil and communicate the transferable knowledge (though more on this might have strengthened this aspect of the bid). The real strength of this work is in the synthesis of many different strands of research to test a practical solution.

3. Quality of the proposed work

Research Design
i) How appropriate is the research design in relation to the stated objectives?
ii) To what extent is the proposed design and methodology for all elements of the research well defined, appropriate, valid and feasible within the timeframe and resources requested?
iii) What are the strengths and weaknesses of the research design as proposed?
iv) To what extent does the research show originality and innovation?

i) The research design is very good relative to the objectives. It is comprehensive, covering the key considerations in designing complex digital health interventions (covering an important range of issues from economics to evaluation). This integration of approaches is necessary if we are to better understand how to deploy novel digital technologies such as chatbots in clinical practice. There are some existing implementations (e.g. Babylon), but the evidence base is currently thin.

ii) The details of some of the design and methodology elements are missing. I will focus on the ones that are within my areas of expertise (since no-one can be an expert on all aspects of such a project). The stated aims in relation to evaluation drift between a focus on patient safety and a focus on acceptability and usability. Patient safety as a theme isn't covered in depth, but the assumption seems to be that clinical assessment is the gold standard. Table one could be summarised in a sentence as "Go with the clinical judgement". Frustratingly, there is no discussion of the decision support algorithm -- i.e., how does DORA reach a decision about review? This might have been addressed in earlier development (getting the system to TRL4), but if the evaluation is to focus on safety then this is a key decision. Also, if many calls are interrupted, that would tell the team a lot about what isn't working about the system, but there is little discussion in the bid about how interruptions will be analysed and responded to (in terms of future system development). Looking beyond the current version of DORA, the intention is that it should function without direct supervision, so it needs to be able to work without interruptions, and there is little discussion of how this transition will be made with confidence. For evaluating user experience, four different questionnaires / theories are proposed. There is no justification of why these have been identified, no account of how they will be used with participants or how a framework such as NASSS will be applied to data. I am broadly very supportive of this proposal, but this is my area of expertise and the proposal is shallow and naive. To do anything meaningful in an evaluation of user acceptance and user experience, the team needs to include someone who actually knows about these things from a research perspective. Elsewhere, the team show intuitive understanding of some of the issues, but (to speak metaphorically) there is a world of difference between someone who understands the theory of gravity and someone who knows that apples fall from trees; this team have demonstrated the latter kind of understanding in relation to user experience rather than the former. I speculate that the same is true in relation to the health economics aspect of the proposal: that is outside my area of experise, but also seems to be presented rather superficially.

iii) The major strengths of this proposal are that it builds on an existing body of research-informed practical experience; that it addresses a real and urgent practical problem (in a way that a more theoretically informed proposal probably wouldn't); that it synthesises necessary insights from a broad range of disciplines; and that it is truly translational and likely to have an impact on practice in the near term. The weakness is that it does this in a way that is naive in relation to several relevant literatures. The major contribution is likely to be to clinical practice rather than any kind of theory.

iv) This research is original and innovative in the synthesis and application of prior developments. That is important.

I have suggested in the rating below that the research hasn't drawn sufficiently on prior literature, but that should not be regarded as a weakness. I think it was Voltaire who said something like "the best is the enemy of the

good", and this proposal embodies great synthesis even if some of the foundations are not explained fully.

Has the research been designed with reference to an appropriate review of the existing literature?
No

4. Strength of the research team

i) How well are the roles of the team members described? Is the overall team well coordinated?<br>ii) On the basis of track record in relevant areas, how qualified are the applicants to undertake the work using the methodologies proposed?<br>iii) To what extent does the research team have the necessary breadth and depth of expertise to deliver the proposed work (*e.g.* as judged by publication output and previous research funding)?<br>iv) How could the strength of the research team be improved?<br>v) If the lead applicant is inexperienced, does he/she have appropriate support (*e.g.* from their organisation and/or more senior colleagues) to deliver the work plan?
i) The roles of all members are described well (even if, as noted earlier, some key skills sets seem a little weak), and the overall team looks strong.<br><br>ii) The applicants are very well qualified in the areas that have brought the project to this point. They appear to be well placed to deliver on the practical aspects of the work. As noted earlier, the plans for evaluation appear naive, and the economic assessment is also under-defined. I would also have welcomed more details on how the conversational agent constructs conversation (i.e. interprets the patient's speech and constructs the next conversational move), but assume that this was covered in earlier projects.<br><br>iii) The team have a good track record of publication in general. Their track record of publishing in areas such as patient safety, user-centred evaluation and health economics seems weaker.<br><br>iv) The team is strong in most areas, but would benefit from additional research expertise in the areas identified above.<br><br>v) The lead applicant has a good track record in practical delivery. The presentation of the proposal gives confidence in the project management and ability to deliver on the work plan.

5. Impact of the proposed work

**Dissemination, outputs and anticipated impact**<br>NIHR aims to fund research that has the potential to be of significant benefit to the NHS, patients and the public. To support this, the applicants should consider how they will achieve impact from the outset. This helps them to identify potential impact and beneficiaries, and plan processes by which the research can directly, or incrementally over time, lead to change.<br><br>i) Have the applicants clearly expressed the problem and outlined how this research contributes towards a solution? Are the planned outputs appropriate and sufficient?<br>ii) Is it clear from the application who or which groups (including, as appropriate, healthcare planners, clinicians, patients and/or policy makers) are expected to benefit the most, how they will be engaged and communicated with, and if appropriate methods of engagement and dissemination are planned?<br>iii) Have the applicants set out appropriate activities and resources to achieve their impact goals? Is there a realistic trajectory and estimate of timescales for the benefit to reach patients/ public/ health and care services? Are there clear connections between outputs, engagement processes and impact goals?

iv) Have the applicants clearly considered what follow-on support they might need to generate or upscale impact, and how they might leverage further investment?<br>v) Have the applicants sufficiently and correctly identified any potential barriers they might face (e.g. IP, regulatory and acceptability) and have they properly considered how these may be overcome?<br>vi) Have the applicants clearly and realistically outlined the anticipated impacts, the likely scale of these impacts (both in the shorter and longer terms), and the sequence in which the impacts might occur?
i) Yes.<br><br>ii) Yes, all looks good and appropriate. The involvement of two NHS trust hospitals (in addition to BHT) is a real strength of the proposal.<br><br>iii) Real-world engagement and impact are very clear and well presented.<br><br>iv) The proposal is excellent in this regard.<br><br>v) Yes. All considered and discussed well.<br><br>vi) The applicants haven't discussed this explicitly in detail, but it seems pretty evident from the proposal and future plans.

6. Value for money

i) Taking into consideration the costs associated with undertaking the research, is there sufficient justification for the resources requested? Are all costs essential for the work proposed?<br>ii) Are appropriate resources set aside for patient and public involvement - including plans for a training and support budget?<br>iii) Where relevant, are resources to support impact - other than those for public and patient involvement - included and appropriate?<br>iv) Taking into account the total cost of the research, including the NHS costs, to what extent does the research provide value for money?<br>v) If required, are funds requested for NHS support and treatment costs appropriate and justified?
i) The costs look appropriate. There is a statement that "The Finance and SoECAT Forms are included as separate documents" but I could not locate those documents. On the basis of the information available, the costs look very reasonable.<br><br>ii) The PPI costs mentioned are "Cost to host PPI meetings and pay expenses." There are more details of plans for engaging PPI, which look well thought through apart from not explicitly discussing training.<br><br>iii) Impact is built in to the heart of the proposal and doesn't need separate resourcing.<br><br>iv) Value for money seems good. This proposal is to fund the next stage of work in an ongoing programme of projects that build on each other. It is an essential stage to move a concept from lab to large scale deployment and the costs for this particular stage seem appropriate.<br><br>v) They appear appropriate, though I haven't located the justification.

7. Involvement of patients and the public

**Was there any patient and public involvement in the**	Yes

**application?**	

i) What is your assessment of the patient and public involvement (if any) in the development of the application, including involvement in: Identifying the research topic; prioritising the research questions; preparing the application (e.g. contributing to the research design); and identifying potential impact?<br>ii) What is your assessment of any proposed plans for patient and public involvement throughout the life of the research? Can you identify particular strengths, weaknesses and/or areas for improvement?
i) The proposers report extensive PPI in developing the technology thus far, and in shaping the direction of the research. While sketchy on details, PPI engagement looks extensive.<br><br>ii) The proposed plans look comprehensive and inclusive. As reading, I was concerned that they might not be planning explicitly to involve people in the demographic most likely to be using DORA, but that was addressed later in the section. They seem to have covered everything (apart from training PPI representatives).

8. Additional Comments

If you have any additional comments, not covered by the sections above, please provide them below.
It is very difficult to get this kind of interdisciplinary research funded because it so often falls down the gaps between funding bodies. But this kind of research is essential if healthcare delivery is to be effective and cost effective in the future. I have noted what appear to be weaknesses in the academic side of this project (in limited details about study design for WP4 & WP5 and in publication plans / track record in those areas) but no proposal of this complexity can be perfect. I consider this project to be a high priority for funding and encourage the team to also work to address the small areas of weakness.

ACCELERATED ACCESS COLLABORATIVE | NIHR | National Institute for Health Research | NHSX

Artificial Intelligence in Health and Care Award Peer Review

Reference Number	AI_AWARD01852
Project Title	Autonomous Telemedicine - Cataract Surgery Follow-up at Two NHS Trusts
Lead Applicant	Dr Nick de Pennington
Host Organisation	Ufonia Limited
Contract Costs	£503,524.00
Reviewer Number	3

Table Of Contents

1. Reviewer Expertise
2. Relevance of the proposed research
3. Quality of the proposed work
4. Strength of the research team
5. Impact of the proposed work
6. Value for money
7. Involvement of patients and the public
8. Additional Comments

1. Reviewer Expertise

**Please indicate the nature of your expertise by clicking on the appropriate tick box(es) below:**
Researcher in a broadly related field

If the tick boxes above do not adequately capture the nature of your expertise, please briefly provide details in the box below (or use it to give us more detail about your expertise if you wish):

2. Relevance of the proposed research

i) How relevant and important is the proposed research to the priorities and needs of the NHS, and does it offer a health/healthcare solution with demonstrable benefit to patients?<br>ii) Does the application demonstrate an awareness and understanding of previous relevant research or developments in this area?<br>iii) To what extent does the proposed research add distinct value or advance existing knowledge in this area, taking into account wider ongoing or completed research?
The direct benefit of the system is convenience for patients as it does not require them to travel to hospital and can take place at the time or duration that suits them if the system will not miss any hospital follow-ups.<br><br>The indirect benefit would be the more free time of clinicians that allows them to provide timely care for patients who need urgent attention.<br><br>This project is informed by a previous safety study the applicant that has shown the prototype works.

3. Quality of the proposed work

**Research Design**<br>i) How appropriate is the research design in relation to the stated objectives?<br>ii) To what extent is the proposed design and methodology for all elements of the research well defined, appropriate, valid and feasible within the timeframe and resources requested?<br>iii) What are the strengths and weaknesses of the research design as proposed?<br>iv) To what extent does the research show originality and innovation?
The research design is comprehensive and aims to deliver all the started objectives. I feel the timeline of the project is quite short: I feel the completion of of the system integration in two hospital in 6 month time would be too ambitious and the time to recruitment of 1000 patients may not be long enough as well. There is no justification why 1000 patients are needed.

Has the research been designed with reference to an appropriate review of the existing literature?
Yes

4. Strength of the research team

i) How well are the roles of the team members described? Is the overall team well coordinated?<br>ii) On the basis of track record in relevant areas, how qualified are the applicants to undertake the work using the methodologies proposed?<br>iii) To what extent does the research team have the necessary breadth and depth of expertise to deliver the proposed work (*e.g.* as judged by publication output and previous research funding)?<br>iv) How could the strength of the research team be improved?<br>v) If the lead applicant is inexperienced, does he/she have appropriate support (*e.g.* from their organisation and/or more senior colleagues) to deliver the work plan?
Most of the team members are clinicians and from the paper mos of them have no experience in leading projects. There is no publication list for any of the applicants, possibly this is a system error. It's hard to assess the expertise of the team with the little info provided. I am worried about the software development and cloud computing task as no one will be able to give the guidance.

5. Impact of the proposed work

**Dissemination, outputs and anticipated impact**<br>NIHR aims to fund research that has the potential to be of significant benefit to the NHS, patients and the public. To support this, the applicants should consider how they will achieve impact from the outset. This helps them to identify potential impact and beneficiaries, and plan processes by which the research can directly, or incrementally over time, lead to change.<br><br>i) Have the applicants clearly expressed the problem and outlined how this research contributes towards a solution? Are the planned outputs appropriate and sufficient?<br>ii) Is it clear from the application who or which groups (including, as appropriate, healthcare planners, clinicians, patients and/or policy makers) are expected to benefit the most, how they will be engaged and communicated with, and if appropriate methods of engagement and dissemination are planned?<br>iii) Have the applicants set out appropriate activities and resources to achieve their impact goals? Is there a realistic trajectory and estimate of timescales for the benefit to reach patients/ public/ health and care services? Are there clear connections between outputs, engagement processes and impact goals?<br>iv) Have the applicants clearly considered what follow-on support they might need to generate or upscale impact, and how they might leverage further investment?

v) Have the applicants sufficiently and correctly identified any potential barriers they might face (e.g. IP, regulatory and acceptability) and have they properly considered how these may be overcome?<br>vi) Have the applicants clearly and realistically outlined the anticipated impacts, the likely scale of these impacts (both in the shorter and longer terms), and the sequence in which the impacts might occur?
It is very clearly stated the potential benefit to patients and how patients will be involved in the study. The other stakeholders such as clinicians and hospitals are not sufficiently engaged, for instance, what are they going to use the time (money) freed up to provide better patients care?<br><br>How to protect the arising IP would be a big question to be answered.<br><br>There is a plan for future funding to support a RCT to demonstrate the value of the solution versus human staff. Given the technical readiness, I am just wondering if the RCT can be done now. this would significantly speed up the commercialisation and adoption process.

6. Value for money

i) Taking into consideration the costs associated with undertaking the research, is there sufficient justification for the resources requested? Are all costs essential for the work proposed?<br>ii) Are appropriate resources set aside for patient and public involvement - including plans for a training and support budget?<br>iii) Where relevant, are resources to support impact - other than those for public and patient involvement - included and appropriate?<br>iv) Taking into account the total cost of the research, including the NHS costs, to what extent does the research provide value for money?<br>v) If required, are funds requested for NHS support and treatment costs appropriate and justified?
I felt this project is still under budgeted. There is a budget for PPI and publication cost. however, there is no cost to cover IP protection such as FTO and patent filing etc and hospital overheads, this may be a potential risk of the project.

7. Involvement of patients and the public

**Was there any patient and public involvement in the application?**	Yes

i) What is your assessment of the patient and public involvement (if any) in the development of the application, including involvement in: Identifying the research topic; prioritising the research questions; preparing the application (e.g. contributing to the research design); and identifying potential impact?<br>ii) What is your assessment of any proposed plans for patient and public involvement throughout the life of the research? Can you identify particular strengths, weaknesses and/or areas for improvement?
This project has excellent PPI. The system development has received input from patients and the current study also provide some useful feedback in designing this project. There are excellent PPI support at Imperial and Oxford and concrete plan to maximise the input from patients and dedicated patient groups.

8. Additional Comments

If you have any additional comments, not covered by the sections above, please provide them below.

ACCELERATED ACCESS COLLABORATIVE

NIHR | National Institute for Health Research

NHSX

Artificial Intelligence in Health and Care Award Public Review

Reference Number	AI_AWARD01852
Project Title	Autonomous Telemedicine - Cataract Surgery Follow-up at Two NHS Trusts
Lead Applicant	Dr Nick de Pennington
Host Organisation	Ufonia Limited
Contract Costs	£503,524.00
Reviewer Number	4

Table Of Contents

1. Reviewer Expertise
2. Relevance of the proposed research
3. Quality of the proposed work
4. Strength of the research team
5. Impact of the proposed work
6. Value for money
7. Involvement of patients and the public
8. Additional Comments

1. Reviewer Expertise

**Please indicate the nature of your expertise by clicking on the appropriate tickbox(es) below:**
Patient or service user with direct experience of this area

If the tick boxes above do not adequately capture the nature of your expertise, please briefly provide details in the box below (or use it to give us more detail about your expertise if you wish):
Public reviewer who additionally needed to seek unscheduled aftercare for complication following their second cataract procedure.

2. Relevance of the proposed research

i) Is there a clear and credible reason for doing this research? If there is, what is it?<br>ii) Is this research important or relevant to patients or carers? Why is that?<br>iii) Could the results of the research make a difference to patients or carers? If yes, how would they make a difference? If not, why not?
i) Yes, the underlying reasons to pursue this line of enquiry, and to develop such an approach utilising Artificial Intelligence (AI) systems were clearly stated, with credible patent/public, and clinician/NHS benefit, e.g. the NHS gains in having an AI telemedicine follow-up dialogue after cataract procedures is presented by the research team as potentially large - reducing up to 90% of current face-to-face out-patient follow-up appointments and time/cost of such; reducing Covid-19 and other potential infection risks through the use of remote contact, increasing staff capacity and enabling them to work to the 'top of their license', and enabling physical space in clinics to be re-focused, or re-allocated. With such potential, and AI developments advancing at speed over time, the opportunity to invest in a technology that will likely become increasing relied on nationally, and internationally, is an argument ably put by the applicant. On the face of it, this research is at the forefront of such initiatives both in this country, and internationally in this clinical domain. AI, it's stated, avoids placing extra burdens into community services if follow-up is moved out of out-patient hospital or equivalent settings; and such use of technology meets NHS Long Term Plan goals in terms of patient benefit, moving away from hospital-based services where possible/acceptable to do so.

The content of the Reference letters shows both the high regard, and need for this team's research.

ii) Yes, the research has a potential to affect the eventual overall uptake of, or reach of routine follow-up appointments after a cataract procedure. Where for instance one Trust's services were cited where approx 72% of patients were able to be follow-up up, I can see the potential for this to rise if the AI platform is demonstrated to be acceptable and as good or better than a clinician face-to-face, thus where a service is under administrative, staffing, resourcing or emergency/unscheduled challenges, patient care can be maintained at a high level and with increased outputs.

iii) Yes, as use of an AI platform for routine follow-up may still foreground patient concerns (and reach people who may otherwise not have received a service, or a timely one), and may enable clinicians to prioritise their time toward people experiencing more serious ophthalmic conditions across the speciality, and/or complications arising from this procedure also.

Additionally, the experience of the AI platform 'learning' from patient dialogue has potential in other NHS areas where routine appointments are required but when they occur there is always the chance a concern or complication may need to be recognised.

The AI platform has an advantage of not being reliant on patient internet access, which increases the potential reach, convenience, and access for patients, and reduces travel requirements which often necessitate other people supporting the patient and/or other services' involvement.

However, there are notes of caution later in this review regarding access to this platform, including cultural/language reach.

3. Quality of the proposed work

Research Design
i) Are the outcomes the researchers are planning to measure appropriate? Will the research ultimately benefit patients, service users, carers and/or the public? Why is that the case? Are there other outcomes that are more important? If so, what are they?
ii) Have the researchers taken a realistic approach to recruiting people to participate in their research? Could this be improved and if so, how? Do you think people are likely to agree to take part? Would you be willing to take part in the research or suggest to a friend that they did?

i) Yes, the outcomes are appropriate, and can additionally be reached in the plans to compare and contrast different pathways patients utilise to seek support eg Oxford A&E due to capacity pressures.

Both 1st and 2nd cataract procedures are included in the study, any differences will be looked for in analysis. This I find very helpful, having had the procedure for both eyes within the space of 14 months.

[It will be interesting to see if differences in lens offer/choice is recorded - I understand the NHS 'default' is to offer distance lens replacement...]

Having an economic analysis within the Work Packages is an important component, and offers a wider relevance for the research, including analysis of NHS tariff implications.

WP4 - an ethics protocol to be developed, and published - a positive inclusion.

ii) It appears appropriate consents taken at the Pre-op assessment for DORA follow-up calls.

Patient acceptability and thus I expect recruitment is being further explored in the team's UK Innovate Grant at

BHT, which adds confidence.

A question arises concerning participant recruitment and access in the this application's research Work Packages:

Access - the applicants state they will be evaluating the conversation model with participants from different populations, cultural backgrounds and accents.

Whilst the ineligibility criteria refreshingly minimal, how will the team be analysing and addressing patients who are in fact ineligible?:

"We note that at present patients with cognitive difficulties, hearing impairment or non-English speakers will not be able to use the system, facing the same limitations they currently do with human telemedicine services. The mitigation for these patient groups is that more complex human delivered interactions (face-to-face, with third parties or with translation) will still be available. In the future product iterations may include multilingual and omnichannel (e.g. text) engagement to support these individuals."

This is arguably a vital area of future development, and current sector-wide concern; particularly in regard also to socio-economic disadvantage which is a further element the team are already investigating.

I noted in the application the team state they have: "...canvassed internationally, AI can cross language and healthcare system boundaries in cataract procedures."

Is there scope to explore this further in the current Packages, to aid future work they potentially propose?

Ultimately, I would be a willing participant, and would suggest to other people to take part in the study. It would help if I was approached, for instance, to know about the PPI opportunities, and the range of opportunities including post-study, that may be available should I be interested; and that such information had been co-produced pre-recruitment in the Work Package.

Has the research team taken account of previous research in this area?	Yes

Work plan and proposed management arrangements
i) How are any plans for patient and public involvement in the research also referred to in the work plan and in the proposed management arrangements? Could the plan and the management arrangements be improved from this perspective?

As I mention later, much of the PPI appears either assumed from the interaction with participants, e.g. Work Package [WP] 2, or if it is in the work plan and the Work Packages, I found I had to assume it would come through from the partnerships and collaborating bodies e.g. WP1: as WP1's focus includes governance agreements and the clinical reference group's monitoring of delivery, I expected to see reference to PPI. Wouldn't Trusts expect formal PPI in such Work Packages, for instance?

WP3 - says local implementation will include the input from patients and professionals - is this co-ordinated PPI? It remains unclear.

WP4 - an ethics protocol is to be developed, and published. A Study Board will be formed. Whilst citing its remit to also ensure adherence to best practice, there is no mention of PPI or PPI representation here.
WP5 (Access and Adoption) - I couldn't find PPI referenced in the AHSN team roles; To support future adoption across the NHS, I think co-ordinated PPI could be a key element for the team to add value to their research.

There is a risk that if PPI is implicit through the interaction of patients with the AI tool, other pertinent, organised PPI framework options may be overlooked, in my view.

Study Advisory Groups are intended to be formed which will have PPI, and key decisions and co-production of research outputs with PPI will occur, but whilst positive moves, the confidence this will be effective is as yet not

present where planning and management of PPI arrangements remain sparse in the application.

Whilst a Lay Representative will be appointed to Project Monitoring Group, it is a Group with a membership predominantly of clinicians and AI team members.
Therefore, it would assist to understand what PPI process there will be for 'appointing' this representative, and what training, support etc will be offered?

A Ufonia PPI group is to be formed, but I found it unclear how specific to this research project it will be, and again, the levels of training and/or support in this technical subject may be on offer, and if little PPI experience, how will that be supported in full?

Additional clarity would assist in understanding the routes and liaison in and out of study Work Package activities and the Project Management Group (PMG), to enable governance scrutiny, evaluation and PPI access to study information.

The intended PPI group and its expansion appears to have Ufonia personnel in key roles e.g. Chair or Co-Chair, inviting thoughts of potential conflicts of interest. This may be an area of concern? It could assist to have wider stakeholder PPI representatives invited to join such a group at an earlier stage, which may the assist dissemination and evaluation?

I appreciate there is a wide-range of PPI-related interests to accommodate, and presenting a more co-ordinated approach within the application would have reassured me that PPI activity was going to have direct effect, rather than my having to assume much of its presence.

Plain English summary
The plain English summary is intended for an interested audience, who are not necessarily specialists. The summary should be written at roughly the same level as an article in a newspaper. With this in mind, please comment on the following:

i) Does the plain English summary give a clear explanation of the research?

- Does it help you carry out your review? If not, why not?
- Is the language used appropriate and clear? If not, where are the problems?
- Are scientific terms, abbreviations and jargon explained? If not, which terms need explanation?

ii) If this research is funded, the plain English summary will be published on a variety of websites, without the rest of this application form. Could this plain English summary be used on its own to describe the proposed research? If not, what further information is needed?

Further information for researchers on how to write a plain English summary and what to include in a summary is available online at NIHR Make it clear https://www.invo.org.uk/makeitclear/.

i) Overall, I thought the plain English summary (PES) required further revision to enable it to be both used on its own to describe the proposed research, and for its inclusion within this application.

Paragraph 3 had a style that I found the most readable, and that carried the desired clarity in its construction, for example.

To assist, paragraph 4 demonstrated I thought the contrast of technical and marketing/partner-orientated language with the plain English summary style of paragraph 3.

I think it would be helpful, and interesting, to know who or what 'Ufonia' is, and its relationship to the NHS?

Acronyms have been inconsistently either explained, or not explained throughout the text, including key terms i.e. 'AI', 'AHSN', STSF'; complex descriptions of processes that a lay person may not necessarily be familiar with not explained, for example: 'AI technology'; 'natural-language AI assistant'; 'telemedicine platform'; 'hackathon'; 'a late phase award', and suchlike.

Terms are not always described straightforwardly, or in the plainest option available, I think, for example, 'synergies'; DORA becomes: 'a solution'; 'relatively digitally disenfranchised populations'; I am not sure if the phrase, 'stereotyped, repetitive', is most apt in a public-facing summary [paragraph 2]?

The term 'clinician' may need defining, and what role they had in the cataract process...would they have been known to patient?

I couldn't find any PPI references or roles/structures mentioned in the PES.

Or PPI in dissemination plans beyond the phrase 'roll-out'.

The study need appeared to emerge from seeking health economy solutions rather than patient request/need.

Given the strengths of this application overall toward patient/public benefit, a lay person-orientated PES is possible to construct, I believe.

4. Strength of the research team

i) Does the research team appear to have the right mix of skills to carry out this research? For example, if the research involves looking at what nurses do, is there a nurse on the team? If not, how could the team be strengthened?
ii) Is there one or more suitably experienced member of the research team with responsibility for coordinating, supporting and delivering patient and public involvement activities? If not, how could this be addressed?
iii) Are patients, service users or carers included in the research team? And if so, is it clear what their role or roles will be and what they will bring to the research team?

i) The core research team appears expert and experienced in this subject area, with international reputation.

No specific patient and public involvement (PPI) Co-Applicant was noted. See ii, below,

Conflicts of Interest:

a. 'The clinical fellows who will supervise the patient calls will be appointed once funding is confirmed. Two members of Ufonia’s team are ophthalmologists and would be suitable candidates if conflicts of interest can be mitigated...'.

It may be that finding independent clinicians to supervise the patient calls would indeed be preferable. It is not made explicit what exactly will otherwise need mitigating if one or both of Ufonia's team ophthalmologists are to be engaged, but their cv's indicate a fair amount of potential areas to be considered I think.

b. As a lay person, I thought the COI statement Re: NdeP a fair one.

c. It wasn't clear if NdeP was to take a lead role in supervising DORA calls to patients.

ii) I could not find specific reference to a research team member with direct responsibility for coordinating, supporting and delivering PPI activities. However, the Lead Applicant as far as I understood, would be present at Project Management Group meetings - possibly as Co-Chair, and this Group will include the Lay Representative. With the appointment of a Project Manager should this application be successful, the research team might consider this post-holder having a lead PPI liaison role and shared oversight of the PPI strategy's development and implementation?

iii) See above.

5. Impact of the proposed work

Dissemination, outputs and anticipated impact

NIHR aims to fund research that has the potential to be of significant benefit to the NHS, patients and the public. To support this, the applicants should consider how they will achieve impact from the outset. This helps them to identify potential benefits and beneficiaries beyond the academic community, and plan processes by which the research can directly or incrementally over time, lead to change in the 'real world'.

i) Have the applicants clearly expressed a real-world problem and how their research contributes towards a 'solution'? How well do the planned outputs match this aim? If not, what changes are needed?
ii) Have the applicants made it clear what impacts they are aiming to achieve from the research? Are these plans appropriate? Are they achievable? Do they seem realistic in terms of scale and timing? If not, what needs changing?
iii) Have the applicants clearly stated who will benefit from this research (e.g., patients, carers, clinicians, policy makers, healthcare planners) and how they will benefit? Are plans to engage and communicate with these individuals/groups appropriate? If not, what is missing?
iv) Have the applicants chosen suitable activities to achieve impact? Have they made it clear how the outputs, beneficiaries and planned impacts are linked? If not, what needs changing?
v) Are the applicants clear on what would be needed (e.g. more funding, further partnerships) to sustain or increase impact after the project? If not, what else needs to be considered?
vi) Have the applicants sufficiently considered the barriers they may face in achieving impact (e.g. regulations, intellectual property/rights, acceptability to users)? Have they adequately considered how to overcome these? If not, what is missing?

i) Yes, the applicants have made a strong case for AI solutions in healthcare settings, utilising an appropriate specialism to design, analyse and develop an intervention. It will additionally act as a 'live' screening or follow-up approach for people post- cataract procedures whilst at the same time refining its functionality through AI natural language learning and practice.

Particularly, an area on on-going patient concern/complaint is addressed by this research - tackling administrative and out-patient appointment backlogs or lack of follow-up services in high-volume specialisms, cataract procedures being an apt example. With the potential gains in time, physical space, re-focused patient care, enabling healthcare professionals to then work at top of their license is also a laudable objective.

The planned outputs do appear to match the study's aims.

The reach or barriers to people from different cultural communities where English is not their first language, might be reconsidered in terms of the ineligibility criteria, given the differences in populations at the two Trusts?

ii) I found the impacts clear, appropriate, achievable. The team has significant experience in this study area, and the application builds on a body of work that gave confidence for these further steps.

iii) Yes, it is clear from the application who the intended beneficiaries are, from patients themselves in terms of ensuring appropriate engagement with clinicians, and healthcare planners through to national policy-making bodies.

Communicating impact, and findings' dissemination did appear to be aimed in the main at the technical/academic/IT sectors, rather than Lay people and their organisations. It was not clear whether the team had fully considered the opportunities and role of PPI in the design and actual delivery of communication and impact dissemination. The PPI strategy if and when co-produced would assist in such considerations, I think.

iv) With the volume of participants called, I would expect the AI natural language learning aspect of the study to provide vital progress. Follow-up post- cataract appointments appear to give a bounded, and practical means to achieve the study's aims, whist offering impact data in healthcare and economic areas without putting at risk patient care or patient options for face-to-face appointments where desirable.

With what in many ways is a clear route to impact through the combination of the Work Packages, it was straightforward for me to understand the necessary linkages internally and externally for the project to proceed to the next step.<br><br>v) Yes, the applicants have made strong partnerships, including with NHS bodies, and have a clear route forward e.g. for a 'late phase award' application following this study. The breadth of supporting studies they have undertaken provides a firm platform for this and future work, with additional international linkage established.<br><br>vi) I did remain unsure of how the access aspects of the study will be integrated - will be evaluating different populations, cultural backgrounds, accents.tackling different pathways.<br><br>I did question whether the rates for complications from cataract procedures were understated in the application?

6. Value for money

The NIHR provides guidance on what can and cannot be included in the costs of research. PMO carries out an initial financial scrutiny of all applications received. A more detailed scrutiny of finances is always carried out on any application that is recommended for funding. For example, see Appendix B of the Applicant Guidance.<br>As a public reviewer, you are not expected to assess whether the entire research budget is costed correctly. However, comments on the following aspects are welcome:<br>i) Overall, does the research budget seem a reasonable investment of public money? Could it save health and social care costs in the long term?<br>ii) Are the resources set aside for patient and public involvement appropriate for the proposed activities? E.g. for training and support, travel and other expenses, staff salaries? For more see: INVOLVE’s Involvement Cost Calculator. If not, how could it be improved?
i) The research team defines this AI development as a 'beachhead' solution - and describe in understandable terms how this AI can then be a platform for other developments - adding value to the investment of public money, freeing-up time and clinical resources for best use. The economic analysis gives the study an opportunity to provide initial costings and comparisons compared to current NHS commissioning costs, and tariffs. It is possible health and social care costs may be reduced in the long term therefore.<br><br>I noted the relatively small business turnover for Ufonia, being mostly grant-based. It would be unfortunate if research gains were ever at risk of being lost or postponed due to business climate circumstances of its SME base.<br><br>ii) It is not possible for me to comment as detailed PPI budget, or other budget items were not supplied with the application documents. In principle, however, a positive note that the applicants stated: 'Payments will be made in recognition of members’ time based on NIHR Involve guidance on payment of fees and expenses for our members actively involved (NIHR, 2013)...and have produced our estimates following use of the Involvement Cost Calculator.'<br><br>As this statement appeared to be attached to the PPI Group [also known as 'Panel - some clarification required here], it would be helpful for this statement to clearly apply across all application areas, and Work Packages.<br><br>If further PPI is developed in the individual Work Packages, analysis and dissemination activities, and exploring the possibility of a PPI Co-Applicant, as suggested elsewhere in this review, then a revised PPI budget and framework may be required. To mitigate this, though, I'm assuming some PPI costs will be borne by collaborators as part of their usual remits?

7. Involvement of patients and the public

**Was there any patient and public involvement in the application?**	Yes

i) What is your assessment of the patient and public involvement in the development of the application including involvement in: identifying the research topic; prioritising the research questions; preparing the application (*e.g.* contributing to the research design); and identifying potential impact?
ii) What is your assessment of any proposed plans for patient and public involvement throughout the life of the research? Can you identify particular strengths, weaknesses and/or areas for improvement?

i) Whilst it remains unclear whether or not patients and public involvement (PPI) occurred in the identification of the original research topic, given the trajectory of subsequent studies that have contributed to this team's application, I think it would be fair to say that the interaction with patients and public in the pilot and acceptability studies that underpin this application was important for refining the research questions.

An example of such an approach would be the pilot conducted in conjunction ten knee post- surgery patients in their proof of concept study - where the text doesn't say if actual PPI in design, analysis etc, but the findings were influential. And I had to assume PPI occurred in translating the patient reported outcome measure (PROM), combined with authors of the Oxford knee score itself.

Another example might be - was there PPI in the analysis and evaluation of iterations toward freezing the version, and the actual frozen version of the AI that will be tested?

The vast majority of dissemination activity appears aimed at technical/academic/IT sectors, and not directly to lay people/public, and their organisations.

ii) It is the lack of clarity, and the need to make assumptions based on prior knowledge of how collaborations might work in such situations that appears to be relied on in this application to detail PPI activity, both prior to, and within the research Work Packages. Whilst I understand that working with a number of partners and forming practical partnerships can make for a complex PPI framework, it may be helpful to consider further the coordination and integration of PPI for its best effect in the PES, Work Packages, analysis, evaluation, impact, and dissemination planning.

It is not apparent an actual PPI plan exists therefore; much is aspirational, and if actioned could well be laudable. But at present, without explicit embedding in Work Packages may remain an aspiration.

It would be helpful to also know what scrutiny and support arrangements will be in place to ensure PPI is effective and having influence.

8. Additional Comments

If you have any additional comments, not covered by the sections above, please provide them below.
